# Awareness and Use of Reiki, Reiki Research, Energy Healing, and Complementary Medicine amongst Australian Health Professionals and the Community

**DOI:** 10.1177/27536130261434249

**Published:** 2026-03-23

**Authors:** Sonia Zadro, Peta Stapleton

**Affiliations:** 1 Faculty of Society and Design, 3555 Bond University, Gold Coast, QLD, Australia

**Keywords:** reiki, awareness, use, attitudes, perceptions, efficacy

## Abstract

Reiki is a non-invasive energy healing therapy originating in Japan. It is believed that a trained therapist can transmit energy to the patient to activate the body’s capacity to heal itself, restore balance, and promote well-being. Despite a growing body of research supporting Reiki’s efficacy, there are few international studies, and no studies in Australia, examining people’s awareness of and use of Reiki. This study aimed to explore the prevalence and awareness of Reiki and energy healing in the Australian population and to see whether these findings reflected recent trends in the use of Complementary and Alternative Medicine (CAM). It also aimed to explore differences between health professions and the Australian community regarding the prevalence and use of this.

This exploratory study used a quantitative cross-sectional design using an online, anonymous survey. From a sample of 457 Australians, nearly all were aware of Reiki (99%) and energy healing (98%), 78% had used energy healing and 69% had used Reiki. This suggests a higher-than-expected level of awareness and use, which may have been influenced by response bias. Despite this, only 43% were aware of any Reiki research, and 73% of the sample held beliefs about research on Reiki’s efficacy that did not align with the current state of Reiki research. This was mainly due to an underestimation of the research on Reiki’s efficacy (30%) or to having “no idea” (28%) of its current state.

Differences between groups were discussed. Medical practitioners used Reiki, energy healing, and CAM less than other professions and the general community. Massage therapists and Nurses used CAM the most, and Massage Therapists used Reiki and Energy Healing the most. Most users of Reiki and energy healing were highly educated. More education about Reiki research and its current status is recommended.

## Background and Rationale for Research

### The Current State of Reiki Research

There is growing evidence for Reiki’s effectiveness. A recent scoping review found 96 peer-reviewed clinical trials of Reiki. Of these, there were 71 peer reviewed RCTs examining the effectiveness of Reiki, with 61 showing only positive significant results or partly positive significant results. Ten RCTs found only non-significant results. No negative results were found.

Promising outcomes for Reiki have been found for anxiety, depression, stress, moderate to high blood pressure, acute and chronic pain, and chronic conditions.^2,3,4,5,6,7,8,9,10,11,12,13,14,15^ Additional RCTs have also found that Reiki produces significant positive outcomes for conditions requiring hospitalisation including cancer patients,^16,17,18,19,20,21,22^ palliative care,^20,23^ pain,^22–27^ cardiac disease,^13^ pulmonary disease,^28^ mothers of hospitalised children,^29^ third molar surgery,^27^ and healthcare professionals.^30,31^

Because Reiki is classified as an alternative biofield therapy (BT) and differs conceptually from the dominant biomedical model, its reported effects are sometimes attributed to placebo mechanisms. However, emerging evidence suggests that Reiki may have effects beyond placebo. In adults, 38 randomised, placebo-controlled studies have evaluated Reiki using reliable, valid outcome measures.^3,5,9,10,11,12,14,22,27,29,32,33,34,35,36,37,38,39,40,41,42,43,44,45,46,47,48,49,50,51,52,53,54,55,56,57,58^ These studies included a placebo-controlled comparison group that received sham Reiki. Of these, 27 reported that Reiki was significantly more effective than placebo on at least one outcome measure.

Many randomised controlled trials (RCTs) of psychological, physical, or energy-based interventions use waitlist or comparison-therapy control groups, which may not fully account for placebo-related effects. The inclusion of sham-controlled conditions in Reiki trials, therefore, represents an approach designed to assess placebo contributions more directly within the RCT framework.

Alternatively, two Cochrane reviews found no or low evidence for Reiki’s effectiveness but appeared to include very few RCTs. A Cochrane SR^32^ of “Complementary and alternative therapies for post-caesarean pain” included a review of Reiki plus analgesia vs analgesia and included only 1 RCT.^33,34^ Another Cochrane SR by Joyce (2015) of Reiki’s impact on depression and anxiety excluded some studies with baseline means in the clinical range,^35,36^ and one study she included used baseline means on the DASS, which were normal for depression and stress and borderline normal for anxiety (Bowden, 2011).

These clinical discrepancies are important because a more recent SR^2^ by the current authors on Reiki’s impact on mental health suggests that Reiki was consistently effective when symptoms were in the clinical range and usually not effective when symptoms were in the normal range. It is to be expected that when treating a problem in the ‘non-clinical range, the ’problem’ is unlikely to respond significantly to treatment because the problem is absent in the beginning. In this SR, 14 RCTs met the inclusion criteria. It concluded that for Reiki’s effectiveness over placebo, there was a high GRADE level of evidence for clinically relevant symptoms of stress and depression, a moderate to high level of evidence for clinically relevant levels of anxiety, a low to moderate evidence for normal levels of stress and burnout, and low evidence for normal levels of anxiety and depression.

In the most highly controlled RCTs of Reiki, Reiki has also shown positive effects on cell cultures, isolated cells, rats, and female dogs. Reiki has also demonstrated statistically significant therapeutic effects over placebo Reiki in all these studies. These studies included a placebo Reiki group despite the expectation that a placebo effect would be highly unlikely, given their focus on non-human living systems. Six such studies demonstrated the following effects of Reiki over placebo: Significantly higher growth of bacteria than controls,^37^ reduced noise-induced microvascular damage in rats,^38^ improved heart-rate homeostasis in rats,^38^ increased survival of directly irradiated cells,^39^ increased photon emission of intervertebral cells in mice and increased collagen 11 and aggrecan (Kent 2020), reduced postoperative pain in female dogs undergoing elective minimally invasive ovario-hysterectomy.^40^ These RCTs provide evidence that Reiki has a therapeutic effect over a placebo in non-human living systems.

While further research is needed, the evidence to date suggests support for Reiki’s efficacy in treating a range of conditions.

### Awareness of Reiki and Reiki Research

There are no studies on the awareness and use of Reiki, or on Reiki research, in Australia, and few studies have examined international awareness of Reiki. A U.S. study of Osteopathic students’ awareness of Asian oriented therapies, including Reiki, found that^41^ approximately half had never heard of Reiki [68]. Another 40% had heard of Reiki but knew little or nothing about it. Only 1 in 10 could explain what Reiki was, and only 2.5% had used it or knew someone who had. Ayurvedic medicine and Reiki were the two Asian-origin CAM practices about which these students knew the least.

A recent Brazilian study^42^ of 783 neurology researchers and/or clinicians found limited awareness of biofield therapy (BT) and related research. Overall, only a third considered BT safe; the remainder were undecided about its safety or considered it unsafe. Only 6.0% believed BT was effective, and the remainder were equally undecided or opposed to its efficacy.

In a recent unpublished Canadian study,^43,44^ 1000 participants were randomly selected and interviewed by phone about the Canadian Health Care System. Overall, only 38% of respondents had heard of BT, including healing touch, Reiki, body talk, Qigong, and energy healing. One-third of respondents said that clear, evidence-based research would encourage them to try BTs. This suggests they were not aware of BT research.

One profession that may have a greater awareness of Reiki and its research is nurses. who have been instrumental in introducing energy-based treatments into the workplace.^45^ For example, nurses established Reiki volunteer programs at the Portsmouth Regional Hospital in New Hampshire, the Reiki Program at Hartford Hospital in Connecticut, the Reiki volunteer program at Brigham and Women’s Hospital in Boston, Massachusetts, and the Mayo Clinic in Minnesota.^45^

#### Prevalence or Use of Reiki

In Australia, there is no research on the prevalence of Reiki, and only limited research on the prevalence of energy healing. One early Australian Survey found that seven per cent of Australians used energy healing in 2005,^46^ and a 2016 Australian survey found that twenty per cent of Australian women aged 31-36 had used prayer and spiritual healing in 2009.^47^ There is also little research internationally. According to a 2002 report estimating CAM use among adults and children in the U.S.,^48^ 1,080,000 adults had used energy healing or Reiki in the previous 12 months; this increased to 1,216,000 in 2007, representing approximately 5%. Similarly, another U.S. study found that, among many other CAM modalities, the prevalence of energy therapies increased from 4% to 9% over the 10-year period from 2002 to 2012 (*P* = 0.03) (Mehta, Hartel et al, 2016). Both these studies suggest that energy healing, like other types of Complementary and Alternative Medicine (CAM), may be increasing. The previously mentioned Canadian study^43^ also assessed the use of BT in Ontario, Canada. Overall, only 6% of respondents had personal experience with BTs or knew someone who did.

Based on this limited research, awareness of Reiki and energy healing may be approximately 30-40%, and prevalence may be approximately 5-10% in a given population. There may also be lower awareness and use among physicians, and possibly higher use among nurses. Given the limited research, any hypotheses are tentative and exploratory. As such, research on the awareness and use of CAM may assist in predicting awareness of and use of Reiki and energy healing.

#### Prevalence of CAM Use in the Community

In Australia, public use of CAM is high and widespread. For example, a Queensland social survey found that from a random sample of 1261 adults, 61.7% had used self-prescribed CAM or visited a CAM practitioner.^49^ An Adelaide study of male cancer outpatients found that 61.5% of respondents had used CAM, and 52.9% were currently using it.^50^ CAM practitioners have also been found to outnumber GPs in four NSW Divisions of General Practice, and in no Division did CAM practitioners number less than half the number of GPs.^51^ Finally, in an Australian longitudinal study of 1835 pregnant women, 48.1% consulted a CAM practitioner and 52% used CAM products (excluding vitamins and minerals) during pregnancy. Other Australian national prevalence rates have found CAM use to be^52^ at two-thirds of the population. Internationally, CAM prevalence rates are also high, with some studies suggesting it is both high and increasing in the U.S.,^53,54^ and high in Europe.^55^

#### Use of CAM by Medical Practitioners

There is little research on CAM use by Australian medical practitioners. An Australia-wide survey conducted in 2010 (Pirotta et al., 2010) compared G.P.’s who used integrative medicine in their practice with those who did not. One-third of respondents identified themselves as practising integrative medicine, 'integrative medicine' included vitamins, minerals, and nutritional supplements, which are now considered by some G.P.’s as mainstream.^56^ Other studies also report negative attitudes toward CAM among medical practitioners, which may explain their more limited use.^42,56^

#### CAM Use by Nurses and Psychologists

A study examining the attitudes of Australian hospital-based nurses towards CAM (Shorofi and Arbon 2017) found that nearly all nurses used CAM for personal use, and about half used CAM professionally. There is also consistent evidence suggesting more positive attitudes toward CAM among nurses, which may indicate higher use within this profession.^57,58,59,60^

A study by Stapleton et al (2015) reported very high use of CAM among psychologists. This study found that, among 193 Psychologists from Australia, the United States, the United Kingdom, and New Zealand, almost all reported using at least one CAM service, and 64.2% were trained to deliver at least one CAM modality. However, another study reported ambivalent attitudes among psychologists in Australia and Indonesia, which may indicate lower CAM use in this profession.^61^

#### CAM Use and Education Level

A number of studies suggest CAM users are the most highly educated members of society.^46,52,62,63,64^ Eisenberg and colleagues first highlighted this in the first systematic national study for CAM use in the US in 1993.^65^ They did a follow-up study of CAM use in the US in 1998.^66^ Among 2055 people in 1997, CAM use in the US was 42%, and it was positively associated with education: 50.6% had some college education compared with 36.4% with no college education (*P* = 0.001). Three recent Australian studies reported similar trends: high CAM use was associated with higher education.^52,63,64^ Given this trend, it would be interesting to examine whether users of energy healing exhibit higher levels of education.

##### Aims of Study 2a

Overall, CAM therapies appear to be widely used and well recognized in Australia and internationally, and research suggests that CAM users tend to be highly educated. There is some evidence that Nurses have greater awareness of and use of CAM and energy healing, and some evidence that medical professionals have less awareness of and use of these modalities. This study explored whether Australian CAM use and awareness were consistent with these findings, and whether Reiki and energy healing followed similar trends. Due to the limited literature on energy healing, Reiki awareness, and its use, this study was also exploratory. Based on these trends in the literature, the following hypotheses were made.

##### Hypotheses

Hypotheses 1 Awareness of Reiki, Energy Healing and Reiki Research

Hypothesis 1a:
Medical practitioners were expected to have the least awareness of Reiki, Energy Healing, and research on Reiki than other professions and community members.
Hypothesis 1b:
Nurses were expected to be more aware of Reiki, Energy Healing, and research on Reiki than other professions and community members.

Hypotheses 2 Usage of CAM, Energy Healing and Reiki

Hypothesis 2a:
Medical practitioners were expected to have used CAM, energy healing, and Reiki less than other professions and community members.
Hypothesis 2b:
Nurses were expected to have used CAM, energy healing, and Reiki more than other professions and community members.

Hypothesis 3 Users of Energy Healing and Reiki were expected to have higher levels of education.

## Method

### Design

The study employed a quantitative cross-sectional design and an online, anonymous survey to assess awareness, prevalence, and attitudes toward Reiki, energy healing, and CAM. This survey was analysed and presented in two studies; this is the first. The second study focused on attitudes. The second paper focused on attitudes. In this study, descriptive statistics, cross-tabulations, chi-square analyses, and One-Way ANOVA were performed using SPSS version 28.0 (2021). All hypotheses were tested using a 5% level of significance.

### Procedure and Materials

The survey *Comparing attitudes towards energy healing and Reiki amongst medical and allied health professionals and the general public* was sent to participants across Australia between the 16^th of^ July 2021 and May 20^th^, 2023. Prior to data collection, ethics approval was obtained from Bond University Human Research Ethics Committee (granted on the 28^th of^ April 2021, BUHREC Number: SZ00068). The participants completed the survey online via Qualtrics. The survey took 10 to 15 minutes to complete, and responses were anonymous and de-identified. Prior to completing the survey, participants were provided with an explanatory statement that included an outline of the study’s purpose.

### Measures

The following questions, related to demographics, questions 7 to 18, asked about a person’s general awareness of and attitudes towards Reiki and energy healing. This was followed by two standardised measures, which were analysed in study 2. Following these two measures, the question “Have you or would you use any of the following treatment options?” was asked. This was followed by fourteen CAM treatment options.

### Demographics

Participants were asked to provide their age, gender, level of education, primary area of employment, and income. If they were a health professional, they were asked to indicate their professional type. Options for health professions included G.P., other medical specialists, dentists, and psychiatrists, and these were all grouped together under “Medical Practitioners” due to low numbers in some categories and all having had a medical training background in common. Nurses were classified as a separate group from “Medical Professionals,” despite some medical training, because the literature suggested they held more positive attitudes toward CAM than doctors and medical specialists. Other categories were Psychologists, Osteopaths/Chiropractors, and Massage Therapists. A high number of respondents chose “other” health profession (*n* = 133). This number increased notably at the time the survey was sent to Reiki Australia, and because the options “energy healer” or “reiki practitioner” were not provided, it was assumed that a number of Reiki Australia members may have selected these options for their profession. As such, those who selected “other” health professional were retained in a separate category, Unidentified Health Professionals (UHP), and it was expected that their attitudes may differ significantly from those of other groups in favour of energy healing and Reiki.

### Participants

Participants were recruited through Facebook, Linked In, and Twitter of professional bodies which included the following: The Australian Medical Association (AMA), The Royal Australian and New Zealand College of Psychiatrists (RANCEP), the Australian Psychological Society (APS), the Australian Health Practitioner Regulation Agency (AHPRA), the Australian Primary Health Care Nurses Association (APNA), the Physiotherapy Board of Australia, the Chiropractic Board of Australia, and the Australian Natural Therapists Association, Reiki Australia, the Australian Reiki connection, Australian Sceptics Incorporated. They were also recruited via word-of-mouth on Facebook. It was difficult to estimate a response rate with such a broad population of potential participants.

Based on a G Power 3.1 for MAC calculator, a sample size of N = 218 was required for the 6 tests run (5 Chi-Square analyses and 1 ANOVA), at a power level of 0.80 and effect size of 0.25 with Chi-Square as the type of test. This was achieved. A total of 508 Australian participants completed the questionnaires; of these, 47 stopped after answering demographic questions. These 47 respondents were deleted, leaving a total N of 461 participants. These 461 participants then answered questions 7 to 18. These questions concerned participants' awareness of and opinions on energy healing and Reiki; all participants’ responses were included in the statistical analysis for these questions. Twenty-three additional participants ceased the survey after question 18, leaving 439 participants. A further 117 respondents initially did not indicate their consent but completed the entire survey. It was assumed this was because it was not a forced response, and they overlooked the consent button. However, consent was presumed because they completed the survey and chose not to terminate. Following the standardised questionnaires, 25 additional participants withdrew, and 414 participants completed the final questions on CAM use and recommendations. Overall, 19% of respondents (*n* = 86) did not report their gender, preventing examination of gender-based prevalence.

Of the 461 participants, 187 were community members, and 274 were health professionals. Of the 274 health professionals, 133 classified themselves as “other health professional” or as mentioned earlier, Unidentified Health Professional (UHP), and 141 identified themselves as one of the following: Psychologist, Nurse, Chiropractor/Osteopath, Physiotherapist, Massage Therapist, G.P, Psychiatrist, Surgeon, Dentist, or other medical specialist. The categories of Psychiatrist, Surgeon, Dentist, G.P., and Other Medical Specialist were re-grouped as the category “Medical Professionals,” creating a total of 23 in this category. The categories of Speech Pathologist 1^1^ and Physiotherapist 3^3^ were excluded due to small group sizes. UHP were analysed as a separate group, with the understanding that this group may contain a higher number of Reiki practitioners.

All groups were classified under a variable labelled “Population” with the final categories as follows: community members (*n* = 187), Psychologist (*n* = 38), Nurse (*n* = 23), Medical Professional (*n* = 23), Osteopath/Chiropractor (*n* = 34), Massage Therapists (*n* = 19), and UHP (*n* = 133).

Respondents came from 22 professions and social sectors and so represented a broad cross-section of society. These included health care (206), retired (88), administration,^19^ Education,^40^ Information Technology,^11^ Medical,^10^ homemaker,^9^ arts and entertainment,^9^ government,^7^ manufacturing,^7^ and finance and insurance.^7^ See Table 1 for group frequencies and demographics. As noted, only 373 participants indicated their gender. No major differences between categories of the “Population” variable were observed for each demographical area: salary, education, age, and gender. For “Demographics,” see Supplementary Material by clicking the link: https://osf.io/qhucs/overview?view_only=d3f66fed1d3740909ab06654dddad447.

**Table 1.** Groups Differences on Reiki Questions With Yes/No Responses. Chi Square Results and Frequencies

Population (valid *N* = 457)									
Question	Chi-square^a^	Community	UHP	Med Prof	Nurse	Psych	Ost/Chiro.	Massage	Total
Frequencies provided for YES responses.	*P* < 0.01	*n* = 187	*n* = 133.	*n* = 23.	*n* = 23	*n* = 38	*n* = 34	*n* = 19	*N* = 457
Have you heard of energy healing, energy medicine or biofield therapy?	NA as nearly 100% answered yes								97.8% (447)
Have you heard of reiki?	NA as nearly 100% answered yes								99.1% (453)
Have you experienced an energy healing treatment?	*P* < 0.001	67.4% (126)	94.7% (126)	34.8% (8)	95.7% (22)	81.6% (31)	64.7% (22)	100% (19)	77.5% (354)
Have you experienced reiki?	*P* < 0.001	57.8% (108)	88.0% (117)	30.4 (7)	95.7% (22)	63.2% (24)	52.9% (18)	100% (19)	68.9% (315)
Are you aware of any reiki research?	*P* < 0.001	32.2% (62)	60.9% (81)	39.1% (9)	65.2% (15)	36.8% (14)	11.8% (4)	68.4% (13)	43.3% (198)

UHP = Unidentified Health Professional, Med Prof = Medical Professional, Psych = Psychologist, Ost/Chiro = Osteopath/Chiropractor.

^a^Chi Sq interpreted with caution as >20% cells (21.4%) have an expected count <5. These cells were in the “No” cells not displayed here.

## Results

### Results for Hypotheses 1: Awareness of Reiki, Energy Healing and Reiki Research

#### Reponses to Yes/No Questions

Cross-tabulation and a Chi-Squared analysis were applied to all “population” variable group responses to Yes/No questions at a 5% level of significance. To identify the degree of awareness of energy healing and Reiki, the following questions were asked: “Have you heard of energy healing, energy medicine, or biofield therapy?” “Have you heard of Reiki?” and “Have you experienced an energy healing treatment?”

Overall, 98% (*N* = 447) had heard of energy healing, energy medicine, or biofield therapy, and 99% (*N* = 453) had heard of Reiki, so no further analysis was done with the presumption there were no significant group differences.

The hypothesis that Medical Professionals would have heard of energy healing/medicine and Reiki the least, and that Nurses and UHP would have heard of it the most, was not supported in this sample.

In response to the question “Are you aware of any Reiki research?” (See Table 1), 43% of participants answered “Yes”. There was a significant difference between groups (*P* < 0.001). Osteo/Chiro scored the lowest with 12% replying with “yes”, followed by 32% of community members, and 37% of Psychologists. The highest-scoring group was Massage Therapists at 68%, followed closely by Nurses at 65% and UHP at 61%.

The first hypothesis, Hypothesis 1a, that Medical Professionals would have the least awareness of Reiki research, was not supported. While Nurses were among the most aware, they were surpassed by massage therapists. Therefore, Hypothesis 1b, that Nurses would be the most aware of Reiki research, was not supported **.**

### Likert Scale Questions

For the question “To what extent do you think Reiki has a sound research base?” (Table 2) the One-Way ANOVA showed that the Levene statistic, the Welch, and the Brown-Forsythe statistic were all highly significant (*P* < 0.001), so the assumption of equality of variances was not met. Because the sample size exceeded 300, absolute values greater than 2 for skewness and greater than 7 for kurtosis were used to assess non-normality. 6^67^ Normality tests indicated that all categories had skewness <2 and kurtosis <7. Histogram plots also appeared reasonable overall, so the assumption of normality was considered met. Because equal variances were not met, Games-Howell post-hoc tests were used to identify individual group differences.

**Table 2.** Likert Response ANOVA Results Does Reiki Have a Sound Research base?

Population (valid *N* = 457)																
Question	Com		UHP		Med Prof		Nurse		Psychol		Ost/Chiro		Massage		Between group *P* value.	Post hoc Sig test results and direction of significance.
	*n* = 187		*n* = 133.		*n* = 23.		*n* = 23		*n* = 38		*n* = 34		*n* = 19			
	*M (SD)*	*MED*	*M (SD)*	*MED*	*M (SD)*	*MED*	*M (SD)*	*MED*	*M (SD)*	*MED*	*M (SD)*	*MED*	*M (SD)*	*MED*		
To what extent do you think reiki as a sound research base?	2.71 (1.37)	3	3.80 (0.98)	4	2.09 (1.35)	2	3.74 (1.18)	4	2.82 (1.14)	3	2.32 (0.98)	3	4.26 (0.56)	4	ANOVA *P* < 0.001	Games-Howell
																Com < Nurse, Mass, UHP.
																UHP and massage > Com, MP, Psych, Ost/Chiro.
																MP < Nurse, Mass, UHP.
																Nurse > Com, MP, Ost/Chiro.
																Psych < Mass, UHP.
																Ost/Chiro < Nurse, UHP, Mass.

Com = Community, UHP = Unidentified Health Professional, Med Prof = Medical Professional, Psych = Psychologist, Mas = Massage Therapist, Ost/Chiro = Osteopath/Chiropractor.

The One-Way ANOVA (See Table 2) revealed a highly significant difference between groups (*f* = 20.81, *P* < 0.001). Medical Professionals had the lowest overall mean of 2.09 (*SD* = 1.35). This suggests their average response was closest to “I believe Reiki may have a small amount of research but is still likely a placebo.” The most common response among medical professionals (48%) was “I believe Reiki has no research base.” This response was selected by 20% of respondents overall and was also rated most highly by the community (30%) and osteopaths/chiropractors (29%). Given the growing evidence to date suggesting Reiki’s effectiveness and that its effects are more than placebo, these results are inconsistent with this.

Post hoc tests revealed that Medical Professionals, Osteopath/Chiropractor, and the community scored significantly lower than Nurses, Massage Therapists, and UHP. As such, although medical professionals held among the lowest beliefs that Reiki has a sound research base, this group was not significantly the lowest overall. Therefore, these findings do not support Hypothesis 1a. Massage therapists and UHPs had significantly higher scores than all other groups, except nurses. Therefore while Nurses had among the highest beliefs that Reiki had a sound research base, they were not significantly the highest overall. As such hypotheses 1b was not supported.

Massage Therapists had the highest mean (4.26, 0.56), followed by UHP (3.80, 0.98) and Nurses (3.74, 1.18). These means are closest to the response “I believe Reiki has a moderate amount of research and is more than a placebo.” This response is more aligned with the current state of Reiki research.

Frequency differences between categories were examined (See Table 3) in answer to this question: “To what extent do you think Reiki has a sound research base?” Overall, 28% of the sample responded with “I have no idea”, closely followed with 27% responding with “I believe Reiki has a moderate amount of research to support its effectiveness and is more than a placebo.” The latter response was rated highest by 63% of Massage Therapists, 40% of UHP, and 35% of Nurses.

**Table 3.** Likert Response Frequencies^a^ Does Reiki Have a Sound Research base?

Population (valid *N* = 457)									
Question	Responses	Community	UHProf.	Med Prof	Nurse	Psych	Ost/Chiro.	Massage	Total
		*n* = 187	*n* = 133.	*n* = 23.	*n* = 23	*n* = 38	*n* = 34	*n* = 19	*N* = 457
1. To what extent do you think reiki has a sound research base to support its effectiveness?	I Believe reiki has no research base	**29.9% (56)**	3% (4)	**47.8% (11)**	4.3% (1)	18.4% (7)	29.4% (10)	0% (0)	19.5% (89)
	I Believe reiki may have a small amount of research but is still likely a placebo	11.2% (21)	5.3% (7)	21.7% (5)	13% (3)	13.2% (5)	14.7% (5)	0% (0)	10.1% (46)
	I Have no idea	27.3% (51)	26.3% (35)	13% (3)	17.4% (4)	**42.1% (16)**	**50% (17)**	5.3% (1)	**27.8% (127)**
	I Believe reiki has a moderate amount of research & is more than a placebo	20.9% (39)	**39.8% (53)**	8.7% (2)	**34.8% (8)**	21.1% (8)	5.9% (2)	**63.2% (12**)	27.1% (124)
	I Believe reiki has a large amount of acceptable research to support its effectiveness & is not a placebo	10.7% (20)	25.6% (34)	8.7% (2)	30.4% (7)	2 (5.3%)	0% (0)	31.6% (6)	15.5% (71)

Highest scores in each category are bolded.

^a^ % given as percentage of number of respondents in that profession/group.

The statement, “I believe Reiki has a large amount of acceptable research to support its effectiveness and is not a placebo,” was never chosen as the most frequent response. However, 16% of respondents overall still chose this option, indicating that a minority overestimated the existing research base.

### Results for Hypotheses 2: Use of CAM, Energy Healing and Reiki

#### CAM Usage

CAM usage was tested by observing different frequency trends in answer to the question: “Have you used or would you use any of the following CAM treatments?” with the 14 CAM treatment options from the CAM health beliefs questionnaire (See Table 4). Of the total respondents, 88% (414) answered this question and reported using or intending to use 3218 types of CAM.

**Table 4.** Prevalence of CAM Usage

Type of CAM	Have you used or would you use the following CAM treatments? (*N* = 414, 87.9% total population)							
	Community *n* = 163	UHP *n* = 125	Med Prof *n* = 19	Nurse *n* = 22	Psych *n* = 36	Ost/Chiro. *n* = 32	Massage *n* = 17	Total (% cases)
Yoga/Meditation/Relaxation	**84.7% (138)**	**98.4% (123)**	**82.4% (16)**	86.4% (19)	**97.2% (35)**	65.6% (21)	88.2% (15)	**88.8% (371)**
Hypnosis	41.7% (68)	56.8% (71)	31.6% (6)	63.6% (14)	50% (18)	46.9% (15)	47.1% (8)	48.6% (203)
Biofeedback	15.3% (25)	26.4% (33)	10.5% (2)	40.9% (9)	30.6% (11)	28.1% (9)	**17.6% (3)**	22.5% (94)
T’ai Chi/Qi Gong	44.2% (72)	64% (80)	36.8% (7)	54.5% (12)	33.3% (12)	43.8% (14)	70.6% (12)	50.5% (211)
Chinese Medicine/Acupuncture/Acupressure	68.7% (112)	80.8% (101)	42.1% (8)	**90.0% (20)**	75% (27)	81.3% (26)	88.2% (15)	74.2% (310)
Ayurveda	30.1% (49)	54.4% (68)	21.1% (4)	45.5% (10)	33.3% (12)	28.1% (9)	64.7% (11)	39.5% (165)
Curanderismo	**2.5% (4)**	**6.4% (8)**	**0% (0)**	**4.5% (1)**	5.6% (2)	**0% (0)**	**17.6% (3)**	**4.3% (18)**
Chiropractic	55.8% (91)	64.8% (81)	36.8% (7)	68.2% (15)	52.8% (19)	**96.9% (31)**	70.6% (12)	61.7% (258)
Massage	**87.7% (143)**	**94.4% (118)**	**68.4% (13)**	**100% (22)**	**83.3 (30)**	**90.6% (29)**	**94.1% (16)**	**89.7% (375)**
Osteopathy	39.3% (64)	56% (70)	26.3% (6)	50% (11)	44.4% (16)	40.6% (13)	70.6% (12)	45.9% (192)
Therapeutic Touch/Reiki	62.6% (102)	89.6% (112)	31.6% (6)	81.8% (18)	55.6% (20)	43.8% (14)	**100% (17)**	69.6% (291)
Spirituality/Prayer	47.2% (77)	68.8% (86)	15.8% (3)	68.2% (15)	**58.3% (21)**	25% (8)	58.8% (10)	53.1% (222)
Herbal/Botanical/Supplements	63.2% (103)	80% (100)	36.8% (5)	77.3% (17)	**58.3% (18)**	71.9% (23)	76.5% (13)	68.7% (287)
Homeopathy	42.3% (69)	70.4% (88)	26.3% (19)	54.5% (12)	50% (36)	53.1% (17)	58.8% (10)	52.9% (221)

Percentages are % of respondents who used energy healing in each category (not % of total responses).

Black bolded scores are the scores with the highest, second highest and lowest frequencies in each group.

The most frequently selected modality was Massage (90%), followed by Yoga/Meditation/Relaxation (89%), and Chinese Medicine/Acupuncture/(74%).

When we look at the highest usage of CAM amongst different professions, 97% Osteopath/Chiropractors used ‘chiropractic’ most frequently. 100% of Massage Therapists selected therapeutic touch/Reiki, and 94% selected massage. Yoga was chosen most frequently by UHP (98%), Psychologists (97%), and Medical Professionals (82%). 100% of nurses and 94% of community members chose massage. All groups chose Massage or yoga/meditation/relaxation as their first or second choice, or both.

Hypothesis 2a that medical practitioners would use CAM less than other groups was supported, as they chose every given category of CAM the least amongst all groups, except for yoga/meditation/relaxation.

Hypothesis 2b, that Nurses would use CAM more frequently than other professionals and community members, was supported. Of 14 CAM modalities, nurses used four of them the most (Massage therapy, Chinese medicine/acupuncture, biofeedback, and hypnosis), two of them the second most (spirituality/prayer, herbal/botanical/supplements), and of the remaining eight CAM modalities, nurses used six the 3^rd^ most frequently. They were comparable however to massage therapists. Massage Therapists used five categories of CAM most frequently (Tai chi/Qigong, Ayurveda, Curanderismo, Osteopathy, Therapeutic Touch/Reiki), three types of CAM second most frequently (homeopathy, chiropractic, Chinese medicine/acupuncture/acupressure), and the remaining ranged from second last to 3^rd^ most.

#### Energy Healing Usage

To identify energy healing usage, trends were examined in response to the question, “If you have experienced an energy healing treatment, which one have you experienced?” Of the total population, 79% (*N* = 361) had experienced an energy healing treatment, and these 361 respondents gave 786 responses indicating which energy healing treatments they had used.

(See Table 5). Additionally, group differences were analysed in response to the yes/no question: “Have you experienced an energy healing treatment?” Cross-tabulation and a Chi-Squared analysis were applied to this yes/no question at a 5% level of significance (See Table 1).

**Table 5.** Responses to “if you Have Experienced Energy Healing Which Type Have you Experienced?”

Type of energy healing	Population *n* = 361 (79% of total *N* = 457) gave 786 responses							
	Community *n* = 130	UHProf *n* = 126	Med Prof *n* = 9 (39%)	Nurse *n* = 22	Psych *n* = 30	Ost/Chiro. *n* = 25	Massage *n* = 19	Total *N* = 361
Reiki	**73.8% (96)**	**86.5% (109)**	**77.8% (7)**	**95.5% (21)**	**60% (18)**	**48% (12)**	**94.7% (18)**	**77.5% (283)**
Therapeutic touch	12.3% (16)	11.9% (15)	0% (0)	22.7% (5)	13.3% (4)	8% (2)	31.6% (6)	13.2% (48)
Qigong	13.1% (17)	25.4% (32)	22.2% (2)	18.2% (4)	13.3% (4)	24% (6)	31.6% (6)	19.5% (71)
Acupuncture	**42.3% (55)**	**51.6% (65)**	22.2% (2)	**36.4% (8)**	**40% (12)**	**52% (13)**	31.6% (6)	**44.4% (162)**
Pranic	10.0% (13)	11.1% (14)	0% (0)	13.6% (3)	3.3% (1)	4% (1)	10.5% (2)	9.6% (35)
Crystal	18.5% (24)	31% (39)	22.2% (2)	27.3% (6)	10% (3)	12% (3)	26.3% (5)	22.5% (82)
Quantum	**4.6% (6)**	**8.7% (11)**	**0% (0)**	**9.1% (2)**	**3.3% (1)**	**0% (0)**	**5.3% (1)**	**5.8% (21)**
Other	17.7% (23)	29.4% (37)	22.2% (2)	22.7% (5)	30% (9)	24% (6)	10.5% (2)	23% (84)

Percentages are % of respondents who used energy healing in each category (not % of total responses).

Black bolded scores are the highest frequency in each profession/group.

Green bolded are the second highest frequency in each profession/group.

Brown bolded are the lowest frequency in each profession/group.

From the total sample (See Table 1) 78% of respondents had experienced energy healing. There was a highly significant difference between the groups (*P* < 0.001). The lowest proportion of respondents who replied ‘Yes’ was Medical Professionals (35%). The next lowest proportion was among osteopaths/Chiropractors (65%). In comparison, 100% of Massage Therapists, followed by 96% of Nurses had experienced energy healing.

Hypothesis 2a, that Medical Professionals would use energy healing the least, was supported. While Nurses were comparable to massage therapists in terms of use, they were slightly lower. Therefore, hypothesis 2b was not supported.

#### Reiki Usage

Amongst users of energy healing only (See Table 5), 78% had used Reiki, and all groups ranked Reiki as their most used energy healing treatment, with 96% of Nurses, 95% of Massage Therapists, and 87% of UHP using it the most. The least commonly used energy healing was Johrei. Because only one person chose Johrei, it was excluded from the cross-tabulation.

In response to “Have you experienced Reiki?” (see Table 1), cross-tabulation and a Chi-Squared analysis were applied at a 5% level of significance. Across the total sample, 69% had received a Reiki treatment, and there was a significant difference between groups (*P* < 0.001). The group with the lowest use overall, was Medical Professionals (30%), followed by Osteopaths/Chiropractors (53%) and community members (58%). Hypothesis 2a, that medical professionals would use Reiki the least, was supported. Those who used Reiki most in the entire sample were Massage Therapists (100%), followed by Nurses (96%). Again, while Nurses were comparable to massage therapists, massage therapists were slightly higher, so hypothesis 2b was not supported.

#### Reiki and Education

Cross-tabulation was also conducted between Education level and use of Reiki and energy healing to assess whether these practices were more prevalent among individuals with higher levels of education. Data for the “Incidence of Energy Healing and Reiki with Education” can be found in Supplementary Material by clicking the link: https://osf.io/qhucs/overview?view_only=d3f66fed1d3740909ab06654dddad447.

In this sample, most users of Reiki and energy healing were highly educated, with over three-quarters of those using energy healing (79%) and three-quarters of those using Reiki (76%) having completed a bachelor’s degree or higher.

## Discussion

### Awareness of Energy Healing, Reiki Research, and the State of Reiki of Research

This study aimed to better identify the level of awareness of energy healing (Reiki) and related research, and the prevalence of Reiki, energy healing, and CAM use in Australia. Additionally, whether awareness of Reiki research aligned with its current research status was explored. It also aimed to determine whether awareness of and use of Reiki differed between community members and health professionals.

Hypotheses 1a, and 1b regarding the awareness of Reiki, Energy Healing and Reiki research were mostly unsupported. Close to all participants in this sample had heard of energy healing (98%) and Reiki (99%), with no difference between groups. This suggests a much higher-than-expected level of awareness of Reiki and energy healing in the wider Australian population than the earlier findings, which reported that 38% of Canadians had heard of BT and 40% of Osteopaths in the U.S. had heard of Reiki.^41,43^

In terms of use, 69% of the sample had used Reiki, and 78% of the sample had used energy healing. This again suggests a much higher prevalence than the limited literature indicates, which reports approximately 5-10% in Australia, Canada, and the U.S.^43,46,48,53^ The findings of a high level of awareness and prevalence in this study likely reflect positive response bias among participants who chose to participate in the survey because they had heard of Reiki. The latter is probable, given that the advertisement to participate stated, “Have your say on complementary medicine and Reiki.” Furthermore, including the terms “energy medicine” and “biofield therapy” in the question “Have you heard of energy healing, energy medicine, or biofield therapy,” could have encouraged ‘yes’ responses from those who had heard of biofield therapy or energy medicine but not necessarily energy healing. Additionally, a higher proportion of respondents from Reiki Australia may have skewed these prevalence rates.

Despite nearly all participants in this sample having heard of Reiki (99%) only 43% of the sample had heard of “any Reiki research”. Given that this sample likely had a disproportionately high level of awareness and use of Reiki, awareness of Reiki research is likely lower in the wider population, suggesting a need for more information about Reiki research. The groups most aware of Reiki research were massage therapists and nurses, with approximately two-thirds of each group reporting awareness. Massage therapists had the highest awareness.

It was also predicted that medical professionals would be the least aware of Reiki research, and this was not supported. The least aware group was Osteopaths/Chiropractors, with 12% aware of Reiki research. However, approximately one-third of the community (32%), Psychologists (37%), and Medical Professionals (39%) were aware of any Reiki research. These findings suggest a need to further promote awareness of Reiki research, particularly among these populations.

In response to the Likert Scale response question, “To what extent do you think Reiki has a sound research base to support its effectiveness?” Medical Practitioners, Osteopaths/Chiropractors, and the community were significantly lower than massage therapists, nurses, and UHP. Twenty percent of the total sample chose “I believe Reiki has no research base.” This included 48% of medical practitioners, 30% of the community, and 29% of Osteopaths/Chiropractors. This estimate is inaccurate and underscores the need for more education about Reiki research, particularly among these professions. Also, 10% chose the response “I believe Reiki may have a small amount of research but is still likely a placebo.” Growing evidence on Reiki’s efficacy over placebo suggests this to also be inaccurate. 28% also responded with “I have no idea.” These responses suggest a need for further education about the current status of Reiki research.

Massage therapists and UHP scored most highly in response to this question. Here, one-third of massage therapists and nurses, and one-quarter of UHP, chose “I believe Reiki has a large amount of acceptable research to support its effectiveness & is not a placebo.” This is likely an overestimation of the research, and these groups may also benefit from further information. These three groups also most frequently chose “I believe Reiki has a moderate amount of research to support its effectiveness and is more than a placebo” at 63%, 35%, and 40%, respectively. This is a closer approximation to the current state of Reiki research, suggesting more awareness amongst these professions.

Overall, these responses suggest that 73% of the sample were likely unaware of the current state of Reiki research, with most underestimating it or reporting they had “no idea,” indicating a need for education in this area.

### Prevalence of Use of CAM, Energy Healing and Reiki

The hypotheses regarding the use of CAM, energy healing, and Reiki were partly supported. In this sample, as hypothesised, Medical Practitioners overall used CAM, energy healing, and Reiki the least. Although nurses were among the highest users of energy healing and Reiki, their use was surpassed by that of massage therapists. Nurses’ use of CAM was also comparable to that of massage therapists. These findings align with the substantial use of CAM therapies among nurses reported in the literature, but they also highlight massage therapists as a leading user group. One possible reason is that, in this sample, some massage therapists may also have been Reiki practitioners, as the two treatments are sometimes combined.

The most frequently used CAM modalities overall were massage and yoga. This is consistent with the literature, as massage is now considered by some as mainstream,^68,69^ and this can skew favourable attitudes towards CAM, as people score in favour of massage because it is no longer viewed as a CAM treatment. Yoga may also be moving in this direction, given its widespread use internationally. There are approximately 300 million yoga practitioners and 20,000 yoga centres worldwide, with Australia having the 3^rd^-highest prevalence, after Singapore and Canada.^70^ As energy healing and Reiki are paradigmatically at odds with the dominant biomedical model, we would expect lower use than other forms of CAM, particularly among medical practitioners. The results of this survey support this idea, as medical practitioners in this sample used energy healing and Reiki less frequently than other professions and community members.

However, in comparison to other types of CAM, Reiki was not the least used CAM treatment overall despite being a more alternative type of CAM. Reiki/Therapeutic Touch is the fourth most used type of CAM (70%), coming just behind Chinese Medicine/Acupuncture/Acupressure (74%).

Hypothesis 3, which posited that users of Reiki and energy healing have high levels of education, was supported. A high level of education among users of Reiki and energy healing was also consistent with CAM research. Over three-quarters of those using energy healing (79%) and Reiki (76%) had completed a bachelor’s degree or higher. This suggests that, alongside CAM users, users of energy healing and Reiki may be more discerning in their use of these practices and are not simply being “duped,” as some critics have argued.^71^

## Strengths

This was the first Australian study to explore the level of awareness and use of Reiki and other forms of energy healing among Australian health professionals and community members. This study identified a high level of awareness and use of Reiki across the sample, with notable differences in awareness of Reiki research and in the prevalence of use between health professionals and community members. It was also the first study to report that, alongside CAM, users of energy healing are more likely to be highly educated. Most importantly, this research identified a lack of awareness of the current state of Reiki research and the professions in which this awareness was most lacking.

The overall sample size (*N* = 457) was robust for an exploratory study, and some findings related to Medical Professionals and Nurses were consistent with the literature on the use of CAM, energy healing, and Reiki. This suggests that the sample may have been representative of the wider population, even if it may have exhibited positive-response bias.

## Weaknesses

Although the total sample was robust, most individual groups were small; therefore, conclusions regarding different professions and the community, while suggestive of trends, cannot be generalized to the wider population. Further research with larger sample sizes for individual groups would help determine whether these findings can be generalised. As noted, 19% of respondents did not indicate their gender, preventing examination of gender-based prevalence.

Chi-squared tests were the primary statistical tests used in this study. Although this shows statistical differences between cells, it does not quantify the strength of the relationship between them. Yes/No closed questions and Likert scale questions were used as opposed to qualitative open-ended questions. This enabled clearer analysis of group differences but limited the interpretations and meanings that could be applied to responses. A regression analysis examining the contributions of age, salary, and education could also have been useful.

Perhaps the most significant flaw in this study was the omission of a separate question to identify respondents who were energy healers or Reiki practitioners. It was presumed that a significant portion of individuals in the category of UHP were energy healers and/or Reiki practitioners. This cannot be verified; therefore, any interpretations of UHP must be treated as tentative. Controlling for practitioners of energy healing in future studies is essential, as this may have confounded results for UHP and potentially for massage therapists, who may have included a higher number of energy healers among their ranks. In future research, steps should be taken to minimise positive response bias from users of energy healing and Reiki. Removing the word “Reiki” from the survey invitation and advertisement may have assisted here. Sampling from a wider population, such as health organisations, hospitals, and the Facebook pages of randomly selected organisations across the country, may also reduce response bias.

## Conclusions

These findings suggest that, in this sample of 457 Australians, nearly all were aware of Reiki and energy healing, and that awareness did not differ between health professionals and community members. This may have been influenced by positive response bias; however, it suggests greater-than-anticipated awareness of Reiki and energy healing among the wider Australian population. Despite this positive bias, only 43% of the sample were aware of any Reiki research. Osteopaths/Chiropractors were the least aware, followed by community members, Psychologists, and medical professionals. Approximately three-quarters of the sample also held beliefs about research on Reiki’s efficacy, which also did not accurately reflect the state of the Reiki research. Most underestimated or had ”no idea” of its state. Medical Professionals, Osteopaths/Chiropractors, and the community were more likely to underestimate the state of Reiki research. Massage therapists, Nurses, and UHPs were more aware of Reiki research and held beliefs more aligned with its current state.

Most respondents reported using energy healing (78%) and Reiki (69%), although this may also reflect a positive response bias. Medical practitioners used Reiki, energy healing, and CAM the least, and massage therapists used Reiki and energy healing the most and were equal to Nurses in CAM use. Most users of Reiki and energy healing were highly educated. This study suggests that, alongside trends in high CAM use, energy healing and Reiki may be well-known and widely used, yet there is limited knowledge of Reiki research and its status. Education about the current status of Reiki research is recommended.

## Supplemental Material

Supplemental Material - Awareness and Use of Reiki, Reiki Research, Energy Healing, and Complementary Medicine amongst Australian Health Professionals and the Community Supplemental Material for Awareness and Use of Reiki, Reiki Research, Energy Healing, and Complementary Medicine amongst Australian Health Professionals and the Community by Sonia Zadro and Peta Stapleton in Global Advances in Integrative Medicine and Health
