# Supplementary material for: Awareness and Use of Reiki, Reiki Research, Energy Healing, and Complementary Medicine amongst Australian Health Professionals and the Community
Source: Glob Adv Integr Med Health. 2026 Mar 23;15:27536130261434249. doi: 10.1177/27536130261434249 (PMC13010006; doi:10.1177/27536130261434249)
Supplement: Supplemental Material - Awareness and Use of Reiki, Reiki Research, Energy Healing, and Complementary Medicine amongst Australian Health Professionals and the Community [file sj-pdf-1-gam-10.1177_27536130261434249.pdf]

Supplementary Material

Demographics

Population Group N=457	**Community**	**Other Health Professionals**	**Medical Professionals**	**Nurse**	**Psychologist**	**Osteopath / Chiropracter**	**Massage therapist**	**Total**
	187 (40.9%)	133 (29.1%)	23 (5%)	23 (5%)	38 (8.3%)	34 (7.4%)	19 (4.2%)	457 (100%)
**Gender**								
	F 87(58%)<br>M 63 (42%)	F 90 (86%)<br>M 14 (13%)	F 10 (45%)<br>M 12 (55%)	F 18 (100%)<br>M (0%)	F 21 (81%)<br>M 5 (19%)	F 11 (32%)<br>M 23 (68%)	F 12 (71%)<br>M 5 (29%)	F 249 (54%)<br>M 122(27%)<br>*86 cases missing
**Age (years)**								
26-35	5.9% (11)	3% (4)	8.7% (2)	0% (0)	13.2% (5)	23.5% (8)	5.3% (1)	6.8% (31)

36-45	9.1%<br>(17)	18%<br>(24)	4.3%<br>(1)	8.7%<br>(2)	28.9%<br>(11)	23.5%<br>(8)	5.3%<br>(1)	14%<br>(64)
46-55	24.1%<br>(45)	27.8%<br>(37)	13%<br>(3)	26.1%<br>(6)	21.1%<br>(8)	14.7%<br>(5)	42.1%<br>(8)	24.5%<br>(112)
56-65	23.5%<br>(44)	40.6%<br>(54)	17.4%<br>(4)	56.5%<br>(13)	26.3%<br>(10)	20.6%<br>(7)	21.1%<br>(4)	29.8%<br>(136)
66-75	26.2%<br>(49)	8.3%<br>(11)	43.5%<br>(10)	8.7%<br>(2)	10.5%<br>(4)	17.6%<br>(6)	26.3%<br>(5)	19%<br>(87)
76-85	8.6%<br>(16)	2.3%<br>(3)	8.7%<br>(2)	0%<br>(0)	0%<br>(0)	0%<br>(0)	0%<br>(0)	4.6%<br>(21)
85 or above	2.1%<br>(4)	0%<br>(0)	4.3%<br>(1)	0%<br>(0)	0%<br>(0)	0%<br>(0)	0%<br>(0)	1.1%<br>(5)
**Highest Level of Education**								
High School	10.7%<br>(20)	6%<br>(8)	4.3%<br>(1)	0%<br>(0)	0%<br>(0)	0%<br>(0)	10.5%<br>(2)	6.8%<br>(31)
Vocational	18.2%	18%	4.3%	8.7%	5.4%	0%	26.3%	14.9%

College	(34)	(24)	(1)	(2)	(2)	(0)	(5)	(68)
Bachelor	24.6%<br>(46)	27.1%<br>(36)	13%<br>(3)	30.4%<br>(7)	2.7%<br>(1)	32.4%<br>(11)	15.8%<br>(3)	23.5%<br>(107)
Post-Grad Diploma	20.9%<br>(39)	28.6%<br>(38)	8.7%<br>(2)	47.8%<br>(11)	8.1%<br>(3)	14.7%<br>(5)	36.8%<br>(7)	23%<br>(105)
Honours	4.8%<br>(9)	2.3%<br>(3)	8.7%<br>(2)	0%<br>(0)	8.1%<br>(3)	0%<br>(0)	10.5%<br>(2)	4.2%<br>(19)
Masters	15%<br>(28)	13.5%<br>(18)	26.1%<br>(6)	13%<br>(3)	54.1%<br>(20)	35.5%<br>(12)	0%<br>(0)	19.1%<br>(87)
Doctorate	5.9%<br>(11)	4.5%<br>(6)	34.8%<br>(8)	0%<br>(0)	21.6%<br>(8)	17.6%<br>(6)	0%<br>(0)	8.6%<br>(39)
**Salary**								
$0 - $40,000	22.5%<br>(42)	23.3%<br>(31)	8.7%<br>(2)	4.3%<br>(1)	7.9%<br>(3)	2.9%<br>(1)	42.1%<br>(8)	19.3%<br>(88)
$40,001 - $60,000	15%<br>(28)	18%<br>(24)	4.3%<br>(1)	4.3%<br>(1)	2.6%<br>(1)	0%<br>(0)	10.5%<br>(2)	12.5%<br>(57)

$60,001 - $80,000	12.3% (23)	9% (12)	8.7% (2)	30.4% (7)	13.2% (5)	5.9% (2)	10.5% (2)	11.6% (53)
$80,001 - $100,000	15% (28)	19.5% (26)	17.4% (4)	17.4% (4)	7.9% (3)	14.7% (5)	10.5% (2)	15.8% (72)
$100,001 - $200,000	22.5% (42)	22.6% (30)	30.4% (7)	39.1% (9)	39.5% (15)	50% (17)	21.1% (4)	27.1% (124)
$200,000 +	12.8% (24)	7.5% (10)	30.4% (7)	4.3% (1)	28.9% (11)	26.5% (9)	5.3% (1)	13.8% (63)

Incidence of Energy Healing and Reiki with Education

	Level of Education						
	High School	Vocational College	Bachelor	Post-Grad Diploma	Honours	Masters	Doctorate
**Those who have used energy healing (N=457)**	6.7% (31)	14.8% (68)	23.5% (108)	23.3% (107)	4.1% (19)	19.1% (88)	8.5% (39)
**Those who have used Reiki (N=317)**	7.3% (23)	17.4% (55)	24.3% (77)	24% (76)	3.5% (11)	18% (57)	5.7% (18)
